# Multistage acupuncture intervention perioperatively: a clinical optimization plan based on the ERAS concept

**DOI:** 10.3389/fmed.2026.1782473

**Published:** 2026-02-19

**Authors:** Jiamin Li, Zhiqiang Dou, Pai Yu, Ting Liu, Yuming Chang, Xingyu Lei, Tie Li, Shuming Zhao, Jiazhen Cao

**Affiliations:** 1 Department of Acupuncture and Tuina, Changchun University of Chinese Medicine, Changchun, Jilin, China; 2 Changchun University of Chinese Medicine, Changchun, Jilin, China

**Keywords:** acupuncture analgesia, acupuncture anesthesia, enhanced recovery after surgery, non- pharmacological intervention, perioperative acupuncture, transcutaneous electrical acupoint stimulation

## 1 Introduction

Post-operative mortality within 30 days of surgery is now the third leading cause of death worldwide, resulting in 3.5 million adult deaths each year and surpassed only by ischemic heart disease and stroke (1, 2). The substantial medical costs, frequent complications, and elevated mortality rates of the perioperative period collectively establish surgical safety as a critical global public health challenge (3). Consequently, developing systematic perioperative management protocols to lower post-operative morbidity and mortality is a priority for clinical teams (4). Enhanced recovery after surgery (ERAS) represents an evidence-based, multimodal strategy that employs standardized pathways to mitigate surgical stress, reduce complications, shorten hospital stays, and control costs (5).

Acupuncture, a cornerstone of traditional Chinese medicine, produces therapeutic effects by stimulating specific acupoints to modulate physiological function. Advances in modern electrophysiology have expanded acupuncture techniques from traditional manual needling and acupressure to include modalities such as electroacupuncture (EA), transcutaneous electrical acupoint stimulation (TEAS), and auricular acupoint electrical stimulation. These innovations have diversified interventional approaches and broadened clinical applications. Perioperative acupuncture can deliver multiple benefits, including pre-operative sedation, intraoperative anesthetic sparing, and post-operative analgesia. Such effects help lower post-operative mortality and adverse event rates, which aligns with the objectives of ERAS (6, 7). Integrating acupuncture with ERAS to optimize management according to patient needs across the perioperative continuum may therefore extend these benefits to a larger patient population.

## 2 Pre-operative: anxiety relief

Pre-operative anxiety is a prevalent psychological issue among surgical patients, typically arising from concerns about surgical outcomes and anesthesia efficacy, approximately 40.5% of patients exhibit high anxiety levels (8). This pathological state excessively activates the sympathetic nervous system, elevating catecholamine levels, stimulating the hypothalamic-pituitary-adrenal axis, and promoting substantial cortisol release (9–11). Such activation induces stress responses, including increased heart rate, elevated blood pressure, and heightened oxygen consumption, which elevate surgical risk and complicate anesthesia management. Persistent anxiety also suppresses immune function, reducing white blood cell and lymphocyte counts while increasing serum levels of pro-inflammatory factors such as IL-1β, IL-6, TNF-α, and IL-2, thereby raising the risk of intraoperative infection and other post-operative complications (12). Severe pre-operative anxiety may further lead patients to delay or cancel surgery, missing the optimal treatment window and adversely affecting long-term quality of life (13).

Current clinical management of pre-operative anxiety relies primarily on pharmacological and non-pharmacological strategies. The variety of medications administered during the perioperative period itself constitutes an independent risk factor for adverse drug reactions (14). Benzodiazepines, for instance, are commonly used for pre-operative anxiolysis but can cause drowsiness and amnesia, potentially prolonging extubation time and impeding early cognitive recovery (15). Consequently, partially or fully replacing pharmacological interventions with effective non-pharmacological methods to reduce medication variety and dosage holds considerable value for patient recovery. As a non-pharmacological therapy, acupuncture alleviates anxiety by activating the vagus nerve, improving autonomic balance through enhanced vagal-sympathetic synchrony, and increasing psychophysiological coherence (16).

Substantial evidence now supports the efficacy and safety of acupuncture for reducing pre-operative anxiety. Both body and auricular acupuncture demonstrate superior anxiolytic effects compared to benzodiazepines, with no significant difference between the two acupuncture modalities (17). Auricular acupuncture, owing to its simplicity and minimal needling points, offers particular clinical utility. Applying finger pressure, Wangbuluxing seeds, or magnetic beads to auricular points within the vagus nerve distribution—such as Shenmen (TF4), Subcortical (AT4), Cardiac (CO15), and Sympathetic Autonomic (AH6a)—effectively lowers heart rate and blood pressure, thereby alleviating pre-operative anxiety (10). Acupoint pressure also reduces anxiety in outpatient surgical patients, facilitating smooth procedures while significantly improving patient satisfaction (18). Adverse reactions like nausea and vomiting occur only rarely. Collectively, acupuncture shows significant efficacy and favorable safety in managing pre-operative anxiety (19). These findings indicate that acupuncture can effectively reduce anxiety, minimize perioperative sedative use, and mitigate associated medication risks while ensuring patient safety.

## 3 Intraoperative: anesthetic support

Anesthesia is critical for ensuring surgical execution and maintaining intraoperative safety, relying on analgesics, sedatives, and muscle relaxants to stabilize vital signs (20). Anesthetic protocols are closely linked to post-operative outcomes: insufficient analgesia can provoke stress responses, disrupt surgery, and hinder recovery, whereas excessive medication may induce neurotoxicity, cognitive impairment, and complications such as post-operative delirium. Optimizing anesthetic strategy—ensuring adequate depth while controlling drug dosage—is therefore vital for improving prognosis. Acupuncture has been employed in surgical anesthesia for over 60 years, yet used alone it often provides incomplete analgesia, muscle tension, and visceral traction (6). The “needle-drug combined anesthesia” model integrates acupuncture with pharmacological anesthesia to address the limitations of each approach alone, achieving complementary and synergistic effects. This method utilizes acupuncture's physiological regulation and analgesic properties to reduce anesthetic drug requirements while maintaining anesthetic depth, thereby lowering drug-related adverse events and offering substantial clinical value (21).

Extensive research shows (6) that acupuncture-assisted anesthesia is applied across diverse surgical fields—including cranial, cervical, cardiothoracic, abdominal, anorectal, orthopedic, and obstetric-gynecological procedures—where it effectively reduces intraoperative sedative and analgesic consumption and mitigates physiological stress associated with conventional anesthesia. Within combined acupuncture-pharmacological anesthesia, TEAS and EA are two commonly used interventions with comparable efficacy (21, 22). In craniotomy, combined acupuncture-pharmacological anesthesia not only reduces intraoperative anesthetic use and accelerates the recovery of spontaneous breathing but also lowers post-operative levels of the brain injury marker S100b, suggesting potential neuroprotective effects (23). Applying TEAS at bilateral Neiguan (PC6), Hegu (LI4), and Zusanli (ST36) points 30 min before anesthesia induction reduces intraoperative remifentanil consumption and decreases post-operative dizziness and pruritus (24). When combined with TEAS, the sedative effect of propofol is enhanced at low concentrations while maintaining stable sufentanil analgesia, offering a novel approach for precise anesthetic management (25). Continued development of needle-drug composite anesthesia has led to significant clinical advances, including successful application in cardiac valve surgery and lung resection without endotracheal intubation. This approach preserves spontaneous breathing and hemodynamic stability while avoiding intubation-related airway injury and post-operative nausea and vomiting (26–28). These explorations demonstrate the clinical value and potential of needle-drug composite anesthesia and justify deeper investigation into its mechanisms.

## 4 Post-operative: effective pain relief

Post-operative acute pain is a common complication of surgical procedures, characterized by severe pain shortly after surgery that impedes early recovery, functional rehabilitation, and overall patient wellbeing. Ineffective management of this pain can hinder short-term recovery and lead to long-term adverse effects. Research shows that myocardial injury following non-cardiac surgery is closely linked to uncontrolled acute pain, with affected patients often demonstrating significantly elevated serum cardiac troponin levels, indicating a correlation between myocardial damage and pain-induced persistent stress responses (21). Beyond organ damage, severe pain also compromises immune defenses, delays wound healing, and elevates the risk of surgical site infection (29). Furthermore, acute pain is a primary factor in the development of persistent post-operative pain, and its inadequate management severely diminishes patients' quality of life (30). Consequently, timely and effective post-operative pain management offers significant clinical benefits by reducing the risk of organ complications, enhancing immune function to prevent infection, and preventing the transition to chronic pain.

A core principle of ERAS is the optimization of pain management. While opioids remain a mainstay for post-operative analgesia in clinical practice, they are suboptimal due to incomplete efficacy and a potential to induce hyperalgesia, thereby exacerbating pain perception (31, 32). Their associated risks of addiction and side effects—including nausea, vomiting, and reduced intestinal motility—can also impede recovery (33–35). Consequently, developing more rational post-operative analgesic strategies is essential. Acupuncture alleviates visceral pain from mechanical traction, spasm, ischemia, or inflammation by modulating central structures and neural circuits across brain regions such as the medulla, cerebral cortex, thalamus, and hypothalamus (36). For neuropathic pain, it acts through multiple pathways, including reducing inflammatory factor release, influencing synaptic plasticity, and regulating brain function (37). Post-operative acute pain arises from multifactorial interactions involving peripheral nociceptor sensitization from surgical trauma, central sensitization, and local ischemic or inflammatory responses. By directly reducing pain intensity and decreasing opioid requirements, acupuncture can indirectly lower the incidence of adverse reactions (32).

Clinical studies confirm the positive role of acupuncture in managing post-operative acute pain. Both traditional filiform needle acupuncture and TEAS reduce pain within the first 24 h after surgery, with TEAS further lowering opioid consumption (34). Given its non-invasive nature, acupuncture exhibits an excellent safety profile and broad acceptability in pediatric populations. TEAS applied at the Neiguan (PC6) and Hegu (LI4) acupoints safely and effectively alleviates post-operative pain in children, reduces analgesic use, improves sleep quality, and significantly increases family satisfaction (38, 39). Effective post-operative pain management should not rely on isolated interventions. Administering repeated acupuncture sessions during the early post-operative period provides superior analgesia compared to single treatments or conventional medication alone (40). Thus, implementing a multi-stage acupuncture protocol spanning the entire perioperative period is crucial for achieving optimal pain relief and preventing chronic pain.

## 5 Key points of clinical acupuncture protocols for the perioperative period

The value of acupuncture in the perioperative period extends beyond providing pre-operative sedation, augmenting intraoperative anesthesia, and relieving post-operative pain. Research confirms its efficacy in preventing and ameliorating common post-operative complications, including nausea and vomiting, gastrointestinal dysfunction, ileus, and post-operative fatigue syndrome, which promotes comprehensive patient recovery (41–45). This aligns closely with the core principle of ERAS, a multimodal, multidisciplinary approach designed to optimize perioperative management. Although numerous studies have been conducted, most have focused on acupuncture interventions in only one or two phases, lacking high-quality research that evaluates the efficacy of comprehensive protocols spanning the entire perioperative period. Consequently, expanding acupuncture application from a single phase to the entire perioperative continuum and integrating it with the established ERAS model to form a unified pre-operative -intraoperative-post-operative intervention system is crucial.

As a pioneer in applying acupuncture to perioperative management, China possesses decades of research foundation and clinical experience in this field. The recent establishment of the National Acupuncture Anesthesia Clinical Research Alliance in Shanghai underscores the significant clinical value and research importance of perioperative acupuncture. This milestone marks the entry of perioperative acupuncture research and application into a phase of standardization. Mirroring the ERAS emphasis on holistic care, perioperative acupuncture targets comprehensive application across all surgical phases. A core mission of this alliance is to explore how acupuncture can be integrated as a fundamental non-pharmacological intervention within the ERAS pathway. By mitigating surgical stress responses and drug-related adverse reactions, this strategy aims to achieve comprehensive optimization of patient outcomes.

Standardized medical protocols are essential for clinical implementation. This section details six critical considerations for the acupuncture component within such protocols.

First, the suitable patient population must be defined, as individual responses to acupuncture vary; establishing scientific inclusion and exclusion criteria is therefore a prerequisite for protocol execution. Following a patient's decision to undergo surgery, a comprehensive condition assessment by professionals, which fully incorporates the patient's preferences, should determine the appropriateness of acupuncture intervention to enhance both its safety and efficacy.

Second, the intervention modality must be selected according to the surgical phase, surgery type, and patient preference, given the diversity of available techniques. Effective perioperative modalities include filiform needle acupuncture, EA, acupressure, auricular acupuncture, and TEAS. Among these, TEAS is notable for its non-invasiveness, ease of implementation, standardization, feasibility of blinding, and minimal interference with surgical procedures.

Third, point selection requires identifying and validating optimal acupoint combinations based on high-level evidence to enhance efficacy. Existing studies have summarized selections for perioperative acupuncture anesthesia (46), such as Yintang (GV24) for pre-operative anxiety, Neiguan (PC6) for post-operative nausea and vomiting, and Shenmen (HT7) for post-operative pain and opioid reduction. Future work can employ data mining to construct standardized acupoint combinations tailored to specific perioperative phases.

Fourth, the timing, frequency, duration, and electrical stimulation parameters (waveform, frequency, intensity) of acupuncture interventions are all critical determinants of therapeutic efficacy, yet clinical application parameters for perioperative acupuncture remain inconsistent. For example, protocols for post-operative pain relief vary widely, including interventions beginning 30 min before surgery and continuing until its conclusion, those applied only after surgery, or a combination of both, with no established consensus on the optimal timing. Future research must therefore prioritize defining optimal parameter sets for different surgical types.

Fifth, during the perioperative period, patients typically receive multiple medications, making it crucial to investigate the interactions between acupuncture and various anesthetics or analgesics. Identifying optimal drug-acupuncture combinations to reduce medication dosage and side effects is essential for developing effective clinical protocols.

Sixth, the efficacy of acupuncture is also highly dependent on the practitioner's expertise and technical skill. Consequently, a comprehensive perioperative management team should include not only surgeons, anesthesiologists, and charge nurses but also licensed acupuncturists with relevant clinical experience. These acupuncturists can contribute to pre-operative assessments to determine a patient's suitability for acupuncture intervention. They can furthermore develop and implement precise acupuncture treatment plans tailored to specific symptoms and individual needs, which helps ensure reliable and consistent therapeutic outcomes.

## 6 Discussion

The safety and efficacy of acupuncture render it a suitable non-pharmacological intervention for perioperative care, with significant benefits demonstrated across all surgical phases. Pre-operatively, it alleviates anxiety, maintains patient stability for smoother surgery, and helps prevent post-operative complications. When applied intraoperatively alongside medication, acupuncture reduces drug dosage, accelerates recovery, and indirectly lowers adverse reaction rates. Post-operative acupuncture promptly relieves acute pain, prevents its transition to chronic pain, and shortens hospital stays. Applying acupuncture comprehensively throughout the perioperative period can prevent or mitigate multiple common complications, producing synergistic effects where the whole exceeds the sum of its parts. In summary, perioperative acupuncture reduces patient suffering, alleviates family anxiety, and improves the utilization of medical resources.

ERAS is a multidisciplinary management model that integrates multiple optimized perioperative measures, ultimately aiming to reduce patient suffering, shorten recovery time, and improve long-term outcomes. Compared to other non-pharmacological ERAS interventions such as music therapy or psychological support, acupuncture offers the distinct advantage of applicability across the entire perioperative timeline. It is effective not only before and after surgery but also provides real-time, continuous stimulation during the procedure itself, demonstrating broad applicability. Peripartum acupuncture reduces the required dosage of sedatives, anesthetics, and analgesics, thereby decreasing drug-related complications and lowering healthcare costs. Furthermore, unlike music or psychological interventions, acupuncture simultaneously regulates both physical and mental states while possessing inherent disease-modifying characteristics. Beyond standard point selection, acupuncture protocols can be adjusted based on individual patient needs to maintain optimal therapeutic effects.

The efficacy of acupuncture involves multiple factors, including patient constitution, practitioner technique, intervention timing, and stimulation parameters, all of which influence the final outcome. Therefore, establishing standardized perioperative protocols through high-quality randomized controlled trials is necessary for clinical guidance. The National Acupuncture Anesthesia Clinical Research Alliance could convene international acupuncture anesthesia and ERAS specialists to develop a professional perioperative acupuncture treatment plan. Subsequent large-scale, multicenter, multinational randomized controlled trials should then be conducted to provide high-quality evidence for clinical implementation.

This paper outlines the clinical application of acupuncture across perioperative phases, proposes an organizational foundation and key considerations for establishing perioperative acupuncture protocols, and advocates for greater recognition of its clinical value by professional teams. Such recognition offers a potential avenue to alleviate patients' financial burdens while reducing post-operative mortality and adverse reaction rates.
